# CT-Based Radiomics in Renal Tumors: Current Evidence, Methodological Challenges, and Future Perspectives for Precision Oncology

**DOI:** 10.3390/cancers18172797

**Published:** 2026-08-28

**Authors:** Anna Colarieti, Sergio Milazzo, Alessandro Pozzo Giuffrida, Alessandro Carriero

**Affiliations:** 1 Department of Translational Medicine, University of Eastern Piedmont, 28100 Novara, Italy; 20068286@studenti.uniupo.it (S.M.); 20068284@studenti.uniupo.it (A.P.G.); alessandro.carriero@med.uniupo.it (A.C.); 2 Radiology Department, Azienda Ospedaliero-Universitaria Maggiore della Carità, 28100 Novara, Italy

**Keywords:** renal cell carcinoma, renal mass, radiomics, computed tomography, machine learning, quantitative imaging, precision oncology

## Abstract

Background: CT-based radiomics may provide non-invasive imaging biomarkers for renal-tumor characterization and risk stratification. This systematic review evaluates the clinical evidence and translational readiness of radiomics in renal oncology and reports an exploratory methodological appraisal of a clearly delimited subgroup. Methods: PubMed/MEDLINE and Embase were searched from database inception through 15 August 2026 using controlled vocabulary and free-text terms for renal tumors, computed tomography, and radiomics or quantitative image analysis. Eligibility was restricted to English-language original studies published from 1 January 2020 to 15 August 2026 that were available in full text. Engineered-feature radiomics constituted the primary evidence base; end-to-end deep-learning studies were considered separately. Owing to clinical and methodological heterogeneity, findings were synthesized narratively. Results: The searches retrieved 1521 records (PubMed/MEDLINE, n = 357; Embase, n = 1164). The PubMed/MEDLINE stream yielded 242 unique records after removal of 115 duplicates; 72 full-text reports were assessed and included. Cross-deduplication and screening of the Embase records identified no additional eligible study. Contemporary evidence supports potential applications in benign–malignant differentiation, histological subtype classification, WHO/ISUP grade and pathological-stage prediction, and postoperative outcome assessment. Reported discrimination was frequently high, but performance estimates were not directly comparable and often declined in independent testing. Within the illustrative, non-representative 28-report detailed appraisal subset, 25 engineered-radiomics studies had an available numerical RQS (median, 16; interquartile range, 15–20; range, 12–24; 44.4% of the maximum). Conclusions: CT radiomics is technically promising but not yet ready for routine clinical use. Quality-related findings apply only to the assessed subgroup and cannot characterize the entire evidence base. Standardized acquisition and feature definitions, transparent analysis, clinically relevant comparators, prospective multicenter validation, formal risk-of-bias assessment in future reviews, and impact studies are needed.

## Simple Summary

Renal masses include benign and malignant lesions with overlapping appearances on conventional computed tomography. Radiomics converts standard images into quantitative descriptors that may support lesion classification and risk stratification. This systematic review critically evaluates recent CT-based radiomics studies in renal tumors, with emphasis on diagnostic discrimination, histological subtype, tumor grade and stage, recurrence, survival, and advanced CT or peritumoral analyses. The reported results are encouraging, but they are difficult to compare because acquisition protocols, segmentation methods, feature engineering, endpoints, and validation strategies vary considerably. Most evidence remains retrospective, and truly independent external testing is still uncommon. Radiomics should therefore be regarded as a promising decision-support approach rather than a replacement for expert imaging assessment or histopathology. Prospective multicenter studies, standardized pipelines, and demonstration of added clinical value are required before routine implementation.

## 1. Introduction

Renal cell carcinoma (RCC) accounts for most malignant renal neoplasms in adults and encompasses histologically and biologically distinct entities, most commonly clear cell RCC (ccRCC), papillary RCC (pRCC), and chromophobe RCC (chRCC). Increased use of cross-sectional imaging has led to more frequent incidental detection of small renal masses, including benign oncocytoma and angiomyolipoma, which may overlap morphologically with malignant tumors [1,2]. Accurate non-invasive characterization is therefore important for selecting active surveillance, biopsy, nephron-sparing surgery, radical nephrectomy, or systemic treatment while avoiding unnecessary intervention.

Multiphasic contrast-enhanced computed tomography (CT) is central to renal-mass assessment because it depicts lesion morphology, enhancement, local extension, venous involvement, and metastatic disease. However, conventional interpretation relies on qualitative and semiquantitative features and may not fully capture spatial heterogeneity. Percutaneous biopsy provides tissue-level information but samples only a limited portion of a heterogeneous tumor and may be nondiagnostic. Quantitative whole-lesion imaging biomarkers could therefore complement—not replace—radiological interpretation and pathology [2].

Radiomics comprises the standardized extraction and analysis of quantitative descriptors of lesion shape, intensity, and texture from medical images [3,4,5]. A typical workflow includes image acquisition, reconstruction and preprocessing, lesion segmentation, feature extraction, feature-reduction and model-selection procedures, internal validation, and, ideally, independent external testing. When integrated with clinical or molecular data, radiomic signatures may characterize phenotypes that are not readily visible to the human eye. The same multistep workflow, however, creates multiple sources of instability and bias.

Recent reviews have emphasized diagnostic classification or selected prognostic endpoints [6,7,8,9]. The field has also expanded rapidly toward dual-energy CT (DECT), peritumoral and perirenal analysis, automated segmentation, deep-learning radiomics, and foundation models. These developments require clear distinction between engineered-feature radiomics and end-to-end representation learning, because their interpretability, validation requirements, and quality-assessment frameworks are not interchangeable.

This systematic review synthesizes CT-based radiomics in renal tumors across four domains: diagnostic characterization; prediction of grade, stage, recurrence, survival, and treatment-related outcomes; advanced CT and extra-tumoral feature analysis; and methodological considerations relevant to clinical translation. All eligible reports contribute to the narrative synthesis. The evidence tables present a purposively selected 28-report subset used solely for illustrative structured mapping. This subset is not representative of the 72-report evidence base, and no subgroup-level characteristic, frequency, or methodological estimate is extrapolated to the complete corpus.

## 2. Materials and Methods

### 2.1. Review Design and Information Sources

This systematic review with predefined eligibility criteria was conducted and reported in accordance with PRISMA 2020 and the PRISMA-S extension [10,11]. The PRISMA checklist can be found in the Supplementary File. The protocol has not been registered. A meta-analysis was not undertaken because the clinical questions, reference standards, cohorts, CT protocols, feature pipelines, and performance measures were too heterogeneous for defensible quantitative pooling. The review was organized around clinically relevant questions rather than pooled effect estimates. PubMed/MEDLINE (PubMed platform) and Embase (Elsevier Embase.com) were searched from database inception through 15 August 2026.

Search strategies combined controlled vocabulary and free-text terms for three concepts: renal tumors, computed tomography, and radiomics or quantitative image analysis. Modeling-related synonyms—including machine learning, artificial intelligence, and predictive models—were included within the radiomics block to maximize sensitivity. Medical Subject Headings and title/abstract terms were used in PubMed; exploded Emtree terms were combined with title, abstract, and author-keyword fields in Embase. No language, date, study-design, or human-only filter was applied at the search stage. Reference lists of pertinent reviews and primary reports were examined for contextually relevant studies. The complete database-specific strategies are provided in Appendix A.

### 2.2. Eligibility Criteria

Eligible primary studies: (i) involved adults with primary renal tumors or renal masses; (ii) evaluated CT-based radiomics, operationally defined as extraction of explicit quantitative image features followed by statistical or machine-learning modeling; (iii) addressed diagnostic characterization, histopathological subtype, grade, stage, invasion, recurrence, metastasis, survival, or treatment-related outcomes; (iv) were published in English between 1 January 2020 and 15 August 2026; and (v) were available as full-text reports. Pathology was considered the preferred reference standard for lesion characterization and histopathological endpoints; time-to-event studies were required to define the outcome and analytical horizon sufficiently for interpretation.

Case reports, series with fewer than 50 patients, animal studies, non-original publications, abstract-only records, purely qualitative imaging studies, segmentation-only studies, radiogenomic studies without an eligible CT-radiomics endpoint, and studies of unspecified renal masses without relevant outcome data were excluded from the primary evidence synthesis. End-to-end convolutional neural networks and foundation-model applications without explicit engineered-feature extraction were classified as adjacent artificial-intelligence approaches and discussed separately. Secondary reviews were used for contextualization only.

### 2.3. Study Selection and Data Extraction

**Figure 1:**
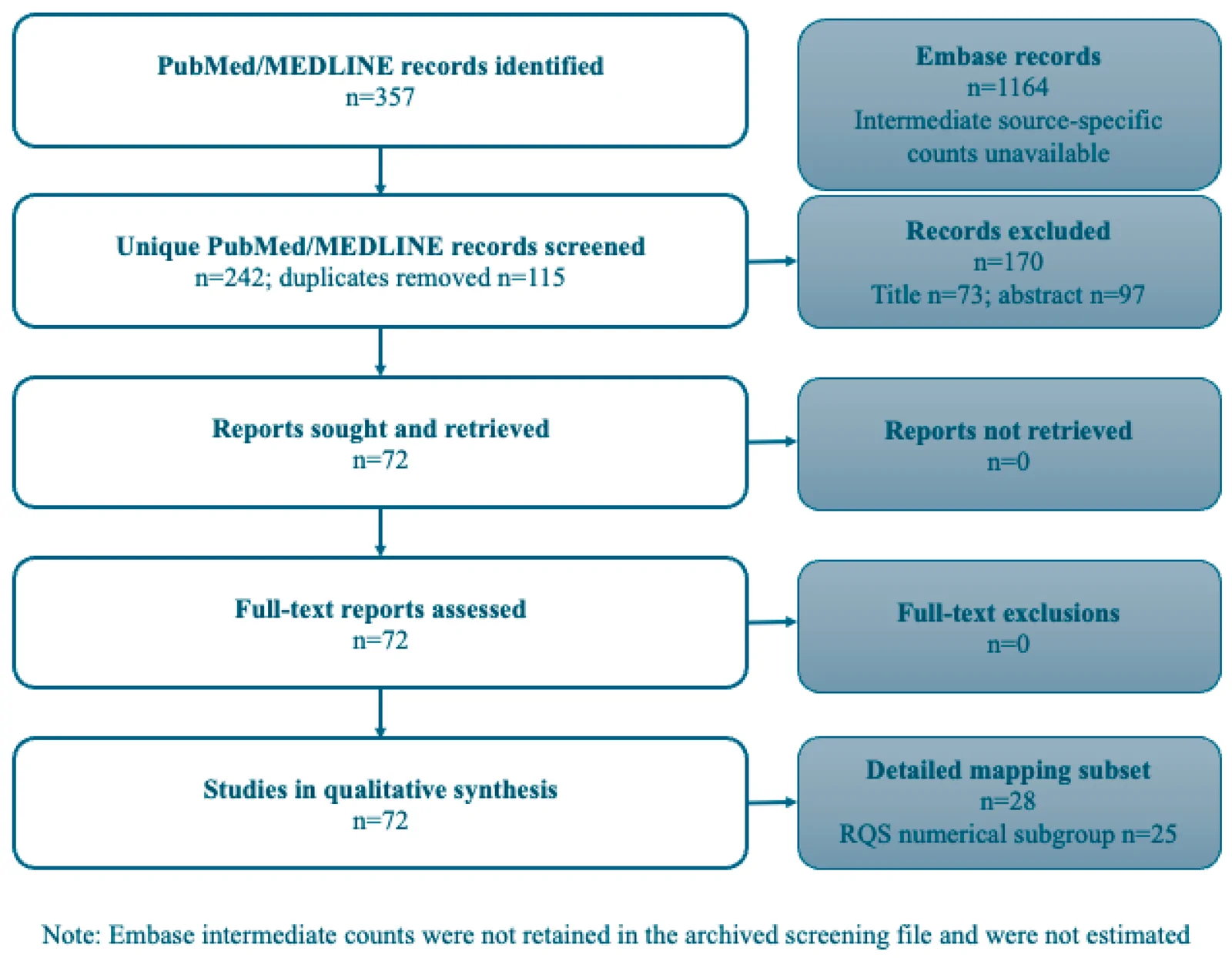
Study-selection flow. The PubMed/MEDLINE stream yielded 72 included full-text studies. Cross-deduplication and screening of 1164 Embase records identified no additional eligible study. The 28-report structured-mapping subset was selected solely for illustrative appraisal and is non-representative; 25 engineered-radiomics studies within that subset had a numerical RQS.

The database searches retrieved 1521 records: 357 from PubMed/MEDLINE and 1164 from Embase. In the PubMed/MEDLINE stream, 115 duplicates were removed, leaving 242 unique records for title/abstract screening. Two radiologists with 3 and 4 years of experience independently screened these records and assessed potentially relevant full texts. Title/abstract screening excluded 170 records (73 by title and 97 after abstract review), leaving 72 reports sought for retrieval. All 72 were retrieved, assessed in full text, and included in the qualitative synthesis; no report was excluded at full-text assessment. The stringent citation-level screen was used to identify reports likely to satisfy all eligibility criteria, with the full text used to confirm eligibility and support data extraction. Embase records were then cross-deduplicated against the PubMed/MEDLINE-derived evidence set and screened using the same criteria; no additional eligible primary study was identified. The archived file does not permit a reliable source-specific breakdown of the intermediate Embase screening counts, which are therefore not estimated. Disagreements were resolved by discussion, with unresolved decisions adjudicated by a senior radiologist with more than 20 years of experience. No abstract-only record contributed to data extraction, evidence synthesis, or methodological appraisal. The selection process is shown in Figure 1.

For each eligible full-text report, the same reviewers independently recorded publication year, study design and setting, sample size, tumor population, clinical endpoint, CT acquisition phase, segmentation and feature-extraction approach, feature-selection method, model type, reference standard, comparator, validation strategy, performance measure, and principal finding. Human-reader number, specialty, and experience were recorded only when explicitly reported. Missing information was designated as not reported and was not inferred. All 72 included reports were synthesized narratively. Tables 1–7 provide detailed structured mapping for 27 reports, and the CT-derived extracellular-volume study [12] is discussed separately as a contextual quantitative-imaging comparator, yielding a 28-report appraisal subset. This subset was selected purposively after eligibility assessment solely to provide illustrative structured mapping of the principal clinical domains, validation patterns, and emerging technical approaches while limiting duplication among studies addressing closely similar questions. It was not selected by a prospectively registered sampling rule, was not random, and is not representative of the full evidence base. The other 44 reports contribute only to the narrative synthesis. Accordingly, no frequency, prevalence, or quality estimate calculated from the 28-report subset is used to characterize all 72 eligible reports.

### 2.4. Evidence Synthesis and Methodological Appraisal

Results were organized by clinical question and synthesized narratively. Performance estimates were reported as presented by the source studies; values from model-development, internal-validation, and independent external-validation cohorts were not pooled or treated as equivalent. Claims of superiority were retained only when a direct comparator and statistical assessment were reported.

Methodological reporting within the 28-report detailed appraisal subset was described with the 16-item Radiomics Quality Score (RQS; maximum 36 points) [4]. Two reviewers assessed the available study-level information independently and resolved discrepancies by consensus, with senior adjudication when required. Twenty-five engineered-radiomics studies provided sufficient information for a numerical RQS; one engineered-radiomics report did not provide sufficient information for a numerical score, and two quantitative-imaging or end-to-end deep-learning reports were outside the RQS summary. Because the RQS is not a risk-of-bias instrument and does not specify a validated dichotomous threshold, no high-/low-quality cut-off was imposed. Scores were summarized using the median, interquartile range, range, and percentage of the maximum. These descriptive scores apply exclusively to the 25 scored studies and were not used to infer the methodological quality or maturity of the remaining studies or of the field as a whole.

### 2.5. Risk-of-Bias Assessment

A dedicated risk-of-bias assessment was not undertaken. PROBAST is designed for studies that develop, validate, or update multivariable diagnostic or prognostic prediction models [13], but the eligible evidence base also included diagnostic classification, prognostic, quantitative-imaging, hybrid, and end-to-end deep-learning designs that were not uniformly amenable to a single tool. In addition, formal risk-of-bias assessment was not prespecified in the review protocol or screening workflow; applying it retrospectively to only a conveniently reportable subset would have introduced another selective appraisal. The RQS was therefore retained solely as a descriptive assessment of radiomics reporting and methodological features in the stated subgroup. This decision limits the review: it cannot provide study-level or evidence-base-level risk-of-bias judgments, and conclusions are restricted accordingly. A future update should prospectively apply PROBAST, or an explicitly justified design-specific tool, to every applicable prediction-model study and report non-applicability separately for other study designs.

## 3. Evidence Synthesis

### 3.1. Evidence Landscape

The initial PubMed/MEDLINE-derived stream yielded 72 eligible full-text reports after deduplication and screening; cross-deduplication and screening of the Embase records added no unique eligible primary study. All 72 reports contributed to the qualitative narrative synthesis (Figure 1). A purposive subset of 28 reports was used solely for illustrative structured mapping, while the remaining 44 contributed only to the narrative synthesis. The mapping subset was not randomly or prospectively sampled and is not representative; its study characteristics, validation patterns, and methodological scores must not be interpreted as estimates for the entire 72-report corpus. Within the full narrative corpus, the literature published from 2020 through mid-2026 included predominantly retrospective reports, alongside an increasing number of multicenter cohorts and independent tests. Contrast-enhanced multiphasic CT was the most common input, although unenhanced CT, DECT iodine maps, and virtual monochromatic images were also evaluated. Manual or semiautomatic three-dimensional segmentation remained prevalent among the reports discussed, while automated segmentation and habitat-based methods appeared increasingly in recent studies.

Most pipelines extracted first-order, shape, and texture features, often after wavelet or Laplacian-of-Gaussian filtering. Reproducibility filtering, least absolute shrinkage and selection operator (LASSO), minimum-redundancy maximum-relevance selection, and correlation-based reduction were common. Logistic regression, support-vector machines, random forests, and gradient-boosting algorithms were frequently used. This technical diversity, together with differences in case mix and validation design, precluded meaningful cross-study ranking based on headline area under the receiver operating characteristic curve (AUC) values.

### 3.2. Diagnostic Characterization

#### 3.2.1. Benign Versus Malignant Renal Masses

Selected studies are summarized in Table 1. Wentland et al. developed a random-forest model from contrast-enhanced CT radiomic features in 148 surgically treated masses; test accuracy was 0.82 (AUC, 0.80), compared with accuracies of 0.67–0.75 for three abdominal radiologists [14]. Wu et al. externally validated single-phase and combined triphasic random-forest models in 427 patients from two centers; external AUCs ranged from 0.821 to 0.921, with the excretory-phase model performing best [15]. Yu et al. analyzed 1795 pathology-confirmed lesions and combined intratumoral, peritumoral, ecological-diversity, and clinical features; the combined model achieved an AUC of 0.946 in a temporally separated test cohort and exceeded the reported reader AUC [16]. These results support further evaluation of potential clinical applicability, but they do not establish clinical utility. The degree of independence between model-development and validation data and the spectrum of benign lesions remain critical determinants of transportability.

Quantitative CT approaches outside the strict engineered-radiomics definition may also be informative. For example, extracellular-volume fraction combined with interpretable machine learning achieved high reported discrimination for small renal masses [12]. Such approaches should be evaluated alongside radiomics, expert interpretation, biopsy strategies, and decision-curve analysis, rather than assumed to be interchangeable.

#### 3.2.2. Histological Subtype and Differential Diagnosis

Histological-subtype studies addressed oncocytoma versus RCC, ccRCC versus non-ccRCC, fat-poor angiomyolipoma versus RCC, and RCC versus upper-tract urothelial carcinoma (Table 2) [17,18,19,20,21,22,23,24,25,26,27,28]. Several models reported AUCs above 0.85, but lesion prevalence, subtype composition, tumor size, CT phases, and reference standards differed substantially. The multicenter oncocytoma–chRCC nomogram reported by Li et al. achieved an external-validation AUC of 0.988 [18], whereas Uhlig et al. showed lower performance when a multicenter subtype classifier was transferred to an external test set [28]. This contrast illustrates why external performance and clinically representative case mix are more informative than the maximum AUC from a development cohort.

A multichannel deep-learning model for fat-poor angiomyolipoma versus RCC achieved an external AUC of 0.898 [26]. Because it learned representations directly from images rather than modeling predefined radiomic features, it is retained in Table 2 as a contextual comparator but excluded from the engineered-radiomics quality summary.

### 3.3. Prognostic and Management Applications

#### 3.3.1. Tumor Grade

Preoperative grade prediction was the most frequently studied prognostic application (Table 3) [29,30,31,32,33,34,35,36,37,38,39,40,41,42,43,44,45,46,47,48]. Investigators evaluated unenhanced, single-phase contrast-enhanced, multiphasic, and DECT data; intratumoral, peritumoral, and habitat-derived features; and manual or automated segmentation. Independent external-validation AUCs reported in the included studies were generally in the mid-to-high 0.80s, with selected habitat or multiphasic models approaching 0.90 [39,42,43]. Nevertheless, grade dichotomization, pathological criteria, class imbalance, and feature-selection procedures varied. A model that separates low- from high-grade ccRCC in a curated surgical cohort may not retain the same calibration or performance in an incidental-mass population, and its clinical utility remains unestablished.

Peritumoral analysis may capture tumor–host interactions that are not represented by the tumor volume alone [30,43]. Automated segmentation could reduce workload and operator dependence, but segmentation performance and downstream model stability should be validated separately [41]. DECT-based signatures have also been linked to grade and microenvironmental markers [44,45,46,47], although scanner and reconstruction dependence may limit immediate portability.

#### 3.3.2. Stage, Invasion, and Surgical Decision Support

Studies of pathological stage and invasion evaluated renal-capsule involvement, perirenal or surrounding-tissue invasion, microvascular invasion, TNM stage, and surgical complexity [49,50,51,52,53,54,55,56,57]. Table 4 highlights an engineered-radiomics model for renal-capsule invasion and a multicenter framework integrating 3D anatomical and radiomic features for nephrectomy planning [54,57]. Other multicenter studies reported gains from intratumoral and peritumoral regions [50,51,52]. The endpoints are clinically relevant, but many analyses used postoperative pathology and selected surgical populations; prospective evaluation is required to show that model output changes management without increasing inappropriate treatment.

#### 3.3.3. Survival, Recurrence, and Metastatic Risk

Radiomic prognostic models have addressed overall survival, recurrence-free survival, distant metastasis, and composite postoperative outcomes [58,59,60,61,62,63,64,65,66,67,68,69,70,71,72,73,74,75,76,77]. Selected studies are shown in Table 5. Reported AUCs or concordance measures were generally promising, but prediction horizons, censoring, follow-up, stage distributions, and clinicopathological comparators varied. Multicenter analyses suggest that radiomic tumor heterogeneity can add information to established prognostic models [61,65,68], although incremental value must be evaluated with calibration, decision analysis, and external validation rather than discrimination alone.

The clinical context is particularly important for recurrence models intended to inform adjuvant-treatment decisions. Models developed in high-risk postoperative cohorts may not generalize to localized low-risk or metastatic disease, and retrospective associations do not establish treatment-effect modification [75,76].

### 3.4. Treatment-Related Prediction and Evidence Gaps

No primary engineered-feature CT-radiomics study represented in the detailed evidence tables evaluated response to systemic therapy as its principal endpoint (Table 6). Prior reviews have discussed treatment outcome and survival more broadly [7], whereas recent primary studies mainly concern recurrence after surgery [75]. Consequently, current evidence should not be interpreted as validating CT radiomics for the selection of a systemic agent or for early response monitoring. Longitudinal, treatment-specific studies with prespecified imaging time points and clinically meaningful reference standards are needed.

### 3.5. Advanced CT, Peritumoral Analysis, and Harmonization

DECT iodine maps, virtual monochromatic images, and peritumoral or perirenal regions expand the biological information available for modeling (Table 7) [44,45,46,47,56,78,79,80]. Studies have associated DECT-derived radiomic features with collagen or interstitial-fiber content and with grade or T stage [45,46,47]. Perirenal adipose-tissue features may reflect local host response and improved grade prediction in a multicenter cohort [79]. These methods are hypothesis-generating, but their dependence on vendor-specific acquisition, reconstruction, and material-decomposition algorithms requires careful cross-platform validation.

Multicenter radiomics is vulnerable to batch effects arising from scanner, reconstruction, and protocol differences. Nested ComBat harmonization improved renal-lesion classification in one multicenter study [81], but harmonization must be fitted without information leakage and validated in genuinely independent data. Standardized feature definitions, such as those promoted by the Image Biomarker Standardisation Initiative, are a prerequisite, but do not by themselves eliminate acquisition-related variability [82].

### 3.6. Adjacent Artificial-Intelligence Approaches

End-to-end deep learning, weakly supervised learning, automated segmentation, and foundation models are increasingly applied to renal CT [26,40,41,42,53,55,77,80,83,84,85,86]. These approaches may offer high predictive performance and reduced dependence on handcrafted features, but they introduce distinct concerns regarding sample size, architecture selection, opacity, domain shift, and reproducibility. They were therefore not pooled conceptually with engineered-feature radiomics. Studies that combine deep features with explicit radiomic and clinical variables should report the contribution and validation of each component.

### 3.7. Methodological Quality

Within the 25 engineered-radiomics studies in the detailed RQS subgroup, the median score was 16 (interquartile range, 15–20; range, 12–24), corresponding to 44.4% of the maximum. This is a descriptive finding for a purposively selected, non-representative subgroup only. It neither constitutes a formal risk-of-bias classification nor supports an estimate of methodological maturity for all included studies or for the field as a whole. Within this subgroup, frequently observed strengths included multivariable modeling and some form of internal validation; frequently observed limitations included retrospective design, limited prospective registration, limited independent external validation, scarce open science, and little evidence of cost-effectiveness or impact on patient outcomes.

Recent multicenter studies represent progress, yet institutional heterogeneity remains consequential. Empirical evaluations of cross-institutional generalizability have demonstrated performance variability even for large deep-learning survival models [77]. Methodological quality should therefore be judged not only by model discrimination, but also by calibration, data provenance, leakage prevention, independent external validation, clinical comparator selection, and transparent reporting.

## 4. Discussion

This review identifies the broad and rapidly evolving literature on CT-based radiomics in renal oncology. Across diagnostic, pathological, and prognostic applications, multiple studies reported clinically interesting discrimination, and several recent multicenter analyses incorporated independent testing. The evidence is nevertheless better interpreted as proof of potential than as validation for routine care. Performance metrics arose from heterogeneous populations, imaging protocols, endpoints, and analytical pipelines; consequently, neither direct cross-study ranking nor a pooled summary estimate would be reliable.

The most immediate diagnostic use case is the distinction of benign from malignant renal masses. Avoiding unnecessary surgery for oncocytoma or angiomyolipoma while maintaining high sensitivity for malignancy has clear clinical value. Studies comparing radiomics with radiologists or integrating peritumoral and clinical information provide encouraging signals [14,15,16]. However, surgical series may be enriched for difficult or higher-risk lesions and can underestimate the spectrum effects encountered in surveillance populations. A clinically useful model should therefore be evaluated at the point of decision, against expert multiphasic CT interpretation and biopsy where relevant, and should report false-negative malignant diagnoses, avoided procedures, calibration, and net benefit.

Subtype and grade prediction are biologically plausible because radiomic features may reflect cellularity, necrosis, vascularity, and spatial heterogeneity. The strongest contemporary studies combine multiphasic or habitat-derived features with clinical variables and perform external validation [18,28,39,42,43]. Yet many models reduce heterogeneous pathological categories to binary outcomes, and differences in grade definitions or lesion composition complicate transportability. Spatial sampling of the entire tumor is an advantage over biopsy, but imaging features remain indirect surrogates and require biological validation rather than post hoc interpretation.

Prognostic applications may be valuable when radiomics adds information to established clinicopathological models. This requirement is more stringent than showing that an imaging signature is associated with an outcome. Models should be compared with validated baselines, evaluated for calibration across clinically relevant time horizons, and assessed with decision-analytic methods. For adjuvant-treatment decisions, prediction of recurrence risk is not equivalent to prediction of treatment benefit. The absence of mature treatment-response evidence in the mapped primary studies is therefore an important gap rather than a neutral finding [7,75,76].

Technical standardization remains central. Slice thickness, reconstruction kernel, contrast timing, dose, scanner platform, interpolation, intensity discretization, segmentation, and software implementation can all alter feature values. Compliance with IBSI definitions [82], detailed protocol reporting, robustness analysis, and locked preprocessing pipelines are necessary. Harmonization can mitigate some batch effects [81] but cannot compensate for biased sampling, inadequate outcome ascertainment, or leakage between training and test sets. Whenever data-driven preprocessing or harmonization is used, it must be fitted within the training data and carried unchanged into validation.

The distinction between engineered radiomics and end-to-end deep learning is clinically and methodologically important. Engineered features may facilitate interpretability and standardized measurement, whereas deep networks can learn task-specific representations but generally require larger datasets and may be more susceptible to domain shift. Hybrid and foundation-model approaches are promising [80,83,84,85,86], but transparent reporting of data provenance, architecture, pretraining, tuning, and external testing is essential. Comparative studies should determine whether these approaches provide reproducible incremental value over simpler models.

A pragmatic translational pathway should proceed from technical validation to clinical validation and then to impact assessment. First, models should use standardized feature computation, prespecified analysis plans, adequate event numbers, and geographically and temporally independent validation. Second, prospective studies should embed the model within the intended workflow, specify how output is interpreted, and compare it with current care. Third, randomized or carefully designed implementation studies should evaluate whether the model improves decisions, patient outcomes, resource use, or cost-effectiveness. Public code, model specifications, and appropriately governed validation datasets would accelerate reproducibility.

This review has limitations. Eligibility was restricted to English-language full-text reports published from 2020 onward, which may introduce language and time-window bias. Although PubMed/MEDLINE and Embase provide broad biomedical coverage, studies indexed only in engineering-oriented databases may not have been captured. The archived screening file did not retain source-specific intermediate Embase counts, preventing full audit of that database stream and precluding reconstruction without unsupported assumptions. The absence of full-text exclusions is unusual but reflects the stringent citation-level screen used before retrieval; the reported zero was retained rather than retrospectively reclassifying exclusions. Substantial clinical and methodological heterogeneity prevented meta-analysis, and reported performance values depend on the source studies’ outcome definitions and validation designs. All 72 eligible reports contributed to the narrative synthesis, but the structured tables map only 27 reports plus one contextual quantitative-imaging comparator [12]. This 28-report subset was purposive, was used solely for illustrative structured mapping, was not selected by a prospectively registered sampling rule, and is not representative of the full 72-report evidence base. The RQS summary applies only to the 25 engineered-radiomics studies with sufficient information for numerical scoring within that subset. No dedicated risk-of-bias tool was applied; therefore, the review cannot make formal risk-of-bias judgments or evidence-base-wide claims about methodological quality.

## 5. Conclusions

CT-based radiomics has the potential to support renal-mass characterization, histological classification, tumor grading and staging, and postoperative risk assessment. Multicenter studies with independent validation and clinically relevant comparators provide the most persuasive signals, but this review did not perform a complete formal risk-of-bias assessment, and its RQS findings concern only a purposively selected subgroup. No conclusion about the overall methodological quality or maturity of the complete 72-report evidence base is therefore warranted. Evidence for systemic-therapy response prediction remains insufficient. Radiomics should remain an investigational decision-support approach pending standardized acquisition and feature definitions, leakage-resistant analysis, transparent reporting, prospective independent external validation, complete design-appropriate risk-of-bias assessment, and prospective demonstration of workflow impact, decision benefit, and patient-level outcomes. Clinical utility cannot be inferred from the predominantly retrospective evidence currently available.

## Figures and Tables

**Table 1:** Selected CT-based radiomics studies addressing differentiation of benign and malignant renal masses.

Reference	Design	n	Lesion/Endpoint	CT Protocol	Radiomics Method	Performance	Validation Category	RQS
Wentland et al. (2023) [14]	Single-center, retrospective	148	Benign vs malignant solid renal masses; comparison with three abdominal radiologists	Contrast-enhanced CT	Tumor segmentation; engineered-radiomic features; random forest; 70:30 train–test split	AUC 0.80; accuracy 0.82. Reader accuracy 0.67–0.75 (*p* = 0.02)	No independent external validation reported	NR
Wu et al. (2024) [15]	Two-center, retrospective	427	Benign vs malignant solid renal tumors	CM, NP, EP	1781 features; mRMR; random forest; phase-specific and combined models; SHAP interpretation	Independent external-validation AUC: CM 0.860; NP 0.821; EP 0.921; combined 0.908	Independent external validation	21
Yu et al. (2024) [16]	Three-site, retrospective	1795	Benign vs malignant renal lesions; whole cohort and small-lesion subgroup	Arterial phase	Intratumoral and peritumoral radiomics; ecological-diversity features; clinical integration	Internal validation AUC 0.946 vs reader AUC 0.823 (*p* < 0.001); small lesions 0.935 vs 0.745	Temporally separated internal validation *	17

* Data were partitioned temporally across three hospital sites, but the report did not describe an independent external-validation cohort. Internal validation: resampling or held-out evaluation within the source population; independent external validation, evaluation in a distinct external population.

**Table 2:** Selected CT-based quantitative imaging studies addressing renal-tumor histological subtype and differential diagnosis.

Reference	Design	n	Lesion/Endpoint	CT Protocol	Radiomics Method	Performance (AUC)	Validation Category	RQS
Li et al. (2022) [18]	Multicenter, retrospective	141	Oncocytoma vs chRCC; CT radiomics nomogram with central scar-matched design	CM, NP, DP	Shape, FO, GLCM, GLRLM, GLSZM, GLDM, NGTDM, wavelet, LoG; LASSO	Clinical model: 0.854 Radiomics model: 0.934 Nomogram: 0.988 (independent external validation)	Independent external validation	24
Han et al. (2022) [25]	Single-center, retrospective	198	Fat-poor AML vs other renal tumors; CT radiomics	UP, CM, NP, EP	Shape, FO, GLCM, GLRLM, GLSZM, GLDM, NGTDM, wavelet	0.83	No independent external validation reported	12
Yu et al. (2022) [20]	Single-center, retrospective	180	Oncocytoma vs ccRCC, pRCC, chRCC (triple-class) + CK7 expression prediction	CM, NP	Shape, FO, GLCM, GLRLM, GLSZM, GLDM, NGTDM, wavelet	Onco vs ccRCC: 0.90 Onco vs pRCC: 0.88 ccRCC vs pRCC: 0.85 CK7: 0.80–0.85	No independent external validation reported	15
Chen et al. (2021) [21]	Single-center, retrospective	197	ccRCC vs non-ccRCC differentiation; multiphase CT radiomics models	UP, CM, NP, EP	296 texture features; LASSO; 3 radiomics models tested across phases	Model 1: 0.748–0.823 Model 2: 0.776–0.887 Model 3: 0.864–0.900	No independent external validation reported	13
Wang et al. (2021) [22]	Single-center, retrospective	190	ccRCC vs non-ccRCC differentiation	CM, NP	396 features; correlation-based selection; RF, LR, SVM classifiers	RF: 0.909 LR: 0.906 SVM: NR	No independent external validation reported	15
Cheng et al. (2023) [23]	Single-center, retrospective	147	ccRCC vs non-ccRCC; radiomics model and nomogram	CM, NP	1130 features extracted; 4 final features via LASSO	>0.949	No independent external validation reported	14
Yao et al. (2023) [26]	Multicenter, retrospective	452	fpAML vs RCC; multichannel DL model on unenhanced CT	UP, NP	Multichannel deep-learning model (not handcrafted radiomics)	Internal validation: 0.966 Independent external validation: 0.898	Independent external validation	NA †
Uhlig et al. (2024) [28]	Multicenter, retrospective	418	Renal-tumor subtype classification (ccRCC, pRCC, chRCC, oncocytoma, AML); multicenter	CM, NP	Shape, FO, GLCM, GLRLM, GLSZM, GLDM, NGTDM; PyRadiomics	Development (venous): 0.81 Independent external validation (venous): 0.75 Independent external validation (combined): 0.71	Independent external validation	20
Marcon et al. (2025) [27]	Single-center, retrospective	236	RCC vs UTUC differentiation on preoperative CT	NP	IBSI-based; shape, FO, GLCM, GLRLM, GLSZM, GLDM, NGTDM; LASSO	RCC vs UTUC: 0.93 (development), 0.87 (internal validation) ccRCC vs high-grade UTUC: 0.84	No independent external validation reported	14

† The Yao et al. model used end-to-end multichannel deep learning and is included for contextual comparison; it was excluded from the engineered-radiomics RQS summary. Development: model-development cohort; internal validation: resampling or held-out evaluation within the source population; independent external validation, evaluation in a distinct external population.

**Table 3:** Selected CT-based radiomics studies for preoperative renal-tumor grade prediction.

Reference	Design	n	Lesion/Endpoint	CT Protocol	Radiomics Method	Performance (AUC)	Validation Category	RQS
Gao et al. (2023) [36]	Single-center, retrospective	113	ccRCC WHO/ISUP grade prediction (tumors < 4 cm); multiphase nomogram	UP, CM, NP, EP	1595 features; PyRadiomics; LASSO	0.940 (model development) 0.902 (internal validation)	No independent external validation reported	14
Zhang et al. (2024) [38]	Single-center, retrospective	198	ccRCC WHO/ISUP grade; stability selection approach	UP, CM, NP, EP	428 features; shape, FO, GLCM, GLRLM, GLSZM, GLDM, NGTDM; stability selection	Single-/combined-phase models: 0.76–0.78	No independent external validation reported	15
Chen et al. (2025) [43]	Multicenter, retrospective	427	ccRCC WHO/ISUP grade; intratumoral + peritumoral habitat imaging	CM, NP	Habitat radiomics; shape, FO, GLCM, GLRLM, GLSZM, GLDM, NGTDM, wavelet	Combined habitat: ~0.92 (model development) ~0.89 (independent external validation)	Independent external validation	22
Wu et al. (2025) [39]	Multicenter, retrospective	169	ccRCC WHO/ISUP grade; multicenter predictive model	CM	FO, shape, texture, high-order; 6 final features (mRMR + LASSO)	Radiomics: 0.873 (development) Combined: 0.895 (development) 0.885 (internal validation), 0.860 (independent external validation)	Independent external validation	21
Yang et al. (2026) [42]	Multicenter, retrospective	377	ccRCC WHO/ISUP grade; DL-based radiomics nomogram (two-center validation)	CM, NP, DP	Shape, FO, GLCM, GLRLM, GLSZM, GLDM, NGTDM + wavelet/LoG	Nomogram: ~0.88 (independent external validation)	Independent external validation	24
Zhu et al. (2026) [41]	Single-center, retrospective	252	ccRCC WHO/ISUP grade; automated DL segmentation vs manual segmentation comparison	CM, NP	Shape, FO, GLCM, GLRLM, GLSZM, GLDM, NGTDM, wavelet	0.80–0.85	No independent external validation reported	20

Internal validation: resampling or held-out evaluation within the source population; independent external validation, evaluation in a distinct external population.

**Table 4:** Selected CT-based radiomics studies for pathological staging, invasion assessment, and surgical decision support.

Reference	Design	n	Lesion/Endpoint	CT Protocol	Radiomics Method	Performance (AUC)	Validation category	RQS
Yang et al. (2022) [57]	Single-center, retrospective	129	Renal-capsule invasion prediction in RCC	CM, NP	Shape, FO, GLCM, GLRLM, GLSZM, GLDM, NGTDM, wavelet	0.80–0.88	No independent external validation reported	15
Yang et al. (2023) [54]	Multicenter, retrospective	473	Surgical decision-making: partial vs radical nephrectomy; 3D-CT anatomical + radiomic features	CM	Traditional radiomic features + anatomical 3D-CT features (p-T and p-G staging)	0.82	No independent external validation reported	17

Independent external validation, evaluation in a distinct external population.

**Table 5:** Selected CT-based radiomics studies for postoperative survival and recurrence prediction.

Reference	Design	n	Lesion/Endpoint	CT Protocol	Radiomics Method	Performance (AUC)	Validation Category	RQS
Wu et al. (2024) [63]	Single-center, retrospective	512	ccRCC postoperative OS prediction (1-, 3-, and 5-year); DL radiomics features; TME and heterogeneity	CM (contrast-enhanced)	3566 DL radiomic features; 2781 reproducible (ICC); LASSO feature selection	1-year: 0.879 3-year: 0.854 5-year: 0.831	Independent external validation	17
He et al. (2025) [67]	Single-center, retrospective	207	Postoperative survival prediction (1- and 2-year) in kidney cancer (various histologies)	Contrast-enhanced CT	5 wavelet features: FO kurtosis, GLSZM, GLRLM, GLDM	1-year: 0.80 (SD 0.03) 2-year: 0.77 (SD 0.04)	No independent external validation reported	16
Feng et al. (2026) [68]	Multicenter, retrospective	194	ccRCC recurrence prediction in high-risk patients post-nephrectomy; tumor heterogeneity + clinicopathological model	CM	Shape, FO, GLCM, GLRLM, GLSZM, GLDM, NGTDM	1-year: 0.882 2-year: 0.865 3-year: 0.793	Independent external validation	20

Independent external validation, evaluation in a distinct external population.

**Table 6:** Evidence gaps in CT-based radiomics for systemic-therapy response prediction.

Clinical Domain	Eligible Primary Studies	Interpretation
Systemic-therapy response prediction	None among the studies selected for detailed evidence mapping	Current renal CT-radiomics evidence primarily concerns diagnosis, pathological characterization, and postoperative prognosis; treatment-specific longitudinal validation remains required.

**Table 7:** Selected CT-based radiomics studies using advanced CT techniques and peritumoral, perirenal, or harmonization approaches.

Reference	Design	n	Lesion/Endpoint	CT Protocol	Radiomics Method	Performance (AUC)	Validation Category	RQS
Li et al. (2023) [45]	Single-center, retrospective	87	ccRCC TME characterization: collagen fiber content prediction using DECT iodine map radiomics	CM, NP (DECT)	2818 features; LASSO; DECT iodine-map derived features	0.722	No independent external validation reported	13
Bing et al. (2024) [46]	Single-center, retrospective	93	ccRCC TME characterization: interstitial fiber evaluation using DECT radiomics	NP (DECT)	2818 features; ICC + variance threshold + SelectKBest + LASSO	Radiomics: 0.973 (development), 0.887 (internal validation) Nomogram: 0.982 (development), 0.918 (internal validation)	No independent external validation reported	15
Wang et al. (2024) [47]	Single-center, retrospective	200	ccRCC nuclear grade + pathological T-staging prediction using DECT radiomics	CM (DECT)	2818 features; shape, FO, GLCM, GLRLM, GLSZM, GLDM, NGTDM; ICC + variance threshold	Nuclear grade: 0.921 (development), 0.882 (internal validation) T-staging: 0.914 (development), 0.875 (internal validation)	No independent external validation reported	16
Ziasaeedi et al. (2025) [81]	Multicenter, retrospective	599	Kidney lesion classification with nested ComBat harmonization for batch-effect mitigation	NP	Shape, FO, GLCM, GLRLM, GLSZM, GLDM, NGTDM; PyRadiomics; nested ComBat normalization	0.950 (with Nested ComBat)	No independent external validation reported	17

Development: model-development cohort; internal validation: resampling or held-out evaluation within the source population; independent external validation, evaluation in a distinct external population.

## Data Availability

The data supporting this review are presented in the article and its tables. Additional extraction materials are available from the corresponding author on reasonable request.

## Supplementary Materials

The following supporting information can be downloaded at: https://www.mdpi.com/article/10.3390/cancers18172797/s1, Supplementary File: PRISMA 2020 Checklist.

## Abbreviations

The following abbreviations are used in this manuscript:

AI	Artificial intelligence
AML	Angiomyolipoma
AUC	Area under the receiver operating characteristic curve
ccRCC	Clear cell renal cell carcinoma
chRCC	Chromophobe renal cell carcinoma
CK7	Cytokeratin 7
CM	Corticomedullary phase
CT	Computed tomography
DECT	Dual-energy computed tomography
DL	Deep learning
DP	Delayed phase
EP	Excretory phase
FO	First-order features
fpAML	Fat-poor angiomyolipoma
GLDM	Gray-level dependence matrix
GLCM	Gray-level co-occurrence matrix
GLRLM	Gray-level run-length matrix
GLSZM	Gray-level size-zone matrix
ICC	Intraclass correlation coefficient
IBSI	Image Biomarker Standardisation Initiative
LASSO	Least absolute shrinkage and selection operator
LoG	Laplacian of Gaussian
LR	Logistic regression
ML	Machine learning
mRMR	Minimum redundancy maximum relevance
MRI	Magnetic resonance imaging
n	Number of patients
NA	Not applicable
NGTDM	Neighboring gray-tone difference matrix
NP	Nephrographic phase
NR	Not reported
OS	Overall survival
pRCC	Papillary renal cell carcinoma
RCC	Renal cell carcinoma
RF	Random forest
RFS	Recurrence-free survival
RQS	Radiomics Quality Score
SHAP	SHapley Additive exPlanations
SVM	Support-vector machine
TME	Tumor microenvironment
TNM	Tumor–node–metastasis
UP	Unenhanced phase
UTUC	Upper-tract urothelial carcinoma
WHO/ISUP	World Health Organization/International Society of Urological Pathology

## Appendix A. Electronic Search Strategies

### Appendix A.1. PubMed/MEDLINE (PubMed Platform)

Coverage: database inception through 15 August 2026. No filters or limits were applied at the search stage.

((“Kidney Neoplasms”[Mesh] OR “Carcinoma, Renal Cell”[Mesh] OR renal tumor*[Title/Abstract] OR renal tumour*[Title/Abstract] OR kidney tumor*[Title/Abstract] OR kidney tumour*[Title/Abstract] OR renal mass*[Title/Abstract] OR kidney mass*[Title/Abstract] OR renal neoplasm*[Title/Abstract] OR kidney neoplasm*[Title/Abstract] OR renal cancer*[Title/Abstract] OR kidney cancer*[Title/Abstract] OR renal cell carcinoma*[Title/Abstract] OR RCC[Title/Abstract] OR ccRCC[Title/Abstract] OR papillary renal cell carcinoma*[Title/Abstract] OR chromophobe renal cell carcinoma*[Title/Abstract] OR renal oncocytoma*[Title/Abstract] OR angiomyolipoma*[Title/Abstract]) AND (“Tomography, X-Ray Computed”[Mesh] OR computed tomograph*[Title/Abstract] OR CT[Title/Abstract] OR contrast-enhanced CT[Title/Abstract] OR multiphasic CT[Title/Abstract] OR dual-energy CT[Title/Abstract] OR spectral CT[Title/Abstract]) AND (radiomic*[Title/Abstract] OR texture analys*[Title/Abstract] OR radiogenomic*[Title/Abstract] OR quantitative imaging[Title/Abstract] OR imaging biomarker*[Title/Abstract] OR image feature*[Title/Abstract] OR feature extract*[Title/Abstract] OR machine learning[Title/Abstract] OR artificial intelligence[Title/Abstract] OR predictive model*[Title/Abstract] OR prediction model*[Title/Abstract] OR classification model*[Title/Abstract] OR nomogram*[Title/Abstract] OR support vector machine*[Title/Abstract] OR random forest*[Title/Abstract] OR logistic regression[Title/Abstract] OR LASSO[Title/Abstract]))

### Appendix A.2. Embase (Elsevier Embase.com)

Coverage: database inception through 15 August 2026. No filters or limits were applied at the search stage.

((‘kidney tumor’/exp OR ‘kidney cancer’/exp OR ‘renal cell carcinoma’/exp OR renal:ti,ab,kw OR kidney:ti,ab,kw) AND (tumor*:ti,ab,kw OR tumour*:ti,ab,kw OR mass*:ti,ab,kw OR neoplasm*:ti,ab,kw OR cancer*:ti,ab,kw OR carcinoma*:ti,ab,kw OR RCC:ti,ab,kw OR ccRCC:ti,ab,kw OR oncocytoma*:ti,ab,kw OR angiomyolipoma*:ti,ab,kw)) AND (‘computed tomography’/exp OR ‘x ray computed tomography’/exp OR ‘computed tomograph*’:ti,ab,kw OR CT:ti,ab,kw OR ‘contrast enhanced CT’:ti,ab,kw OR ‘multiphasic CT’:ti,ab,kw OR ‘dual energy CT’:ti,ab,kw OR ‘spectral CT’:ti,ab,kw) AND (radiomic*:ti,ab,kw OR ‘texture analys*’:ti,ab,kw OR radiogenomic*:ti,ab,kw OR ‘quantitative imaging’:ti,ab,kw OR ‘imaging biomarker*’:ti,ab,kw OR ‘image feature*’:ti,ab,kw OR ‘feature extract*’:ti,ab,kw OR ‘machine learning’/exp OR ‘artificial intelligence’/exp OR ‘machine learning’:ti,ab,kw OR ‘artificial intelligence’:ti,ab,kw OR ‘predictive model*’:ti,ab,kw OR ‘prediction model*’:ti,ab,kw OR ‘classification model*’:ti,ab,kw OR nomogram*:ti,ab,kw OR ‘support vector machine*’:ti,ab,kw OR ‘random forest*’:ti,ab,kw OR ‘logistic regression’:ti,ab,kw OR LASSO:ti,ab,kw)
